# Amebic liver abscess with bronchopleural fistula

**DOI:** 10.1093/jtm/taag006

**Published:** 2026-01-28

**Authors:** Michaela Schumacher, Andrea Erba, Seraina Bally, Seraina Frei, Maja Weisser Rohacek, Andreas Neumayr, Anne-Valérie Burgener-Gasser

**Affiliations:** Division of Infectious Diseases, University Hospital Basel, Petersgraben 4, Basel 4031, Basel Stadt, Switzerland; Division of Infectious Diseases, University Hospital Basel, Petersgraben 4, Basel 4031, Basel Stadt, Switzerland; Division of Infectious Diseases, University Hospital Basel, Petersgraben 4, Basel 4031, Basel Stadt, Switzerland; Division of Intensive Care Medicine, University Hospital Basel, Petersgraben 4, Basel 4031, Basel Stadt, Switzerland; Division of Infectious Diseases, University Hospital Basel, Petersgraben 4, Basel 4031, Basel Stadt, Switzerland; Swiss Tropical and Public Health Institute, Aeschenplatz 2, Basel 4052, Basel Stadt, Switzerland; University of Basel, Petersplatz 1, Basel 4051, Switzerland; College of Medicine and Dentistry, Division of Tropical Health and Medicine, James Cook University, 1 James Cook Drive, Townsville, Queensland 4814, Australia; Division of Infectious Diseases, University Hospital Basel, Petersgraben 4, Basel 4031, Basel Stadt, Switzerland; Division of Clinical Epidemiology, Department of Clinical Research, University Hospital Basel, Totengässlein 3, Basel 4051, Switzerland

**Keywords:** Sepsis, rapid molecular diagnostic, returning traveller, extraintestinal amebiasis, intestinal amebiasis

## Abstract

Amebic liver abscess with bronchopleural fistula caused by *Entamoeba histolytica* can mimic severe bacterial sepsis in a returning traveller. Rapid diagnosis via multiplex Polymerase Chain Reaction (PCR) on abscess fluid enabled targeted therapy within 24 h, avoiding unnecessary broad-spectrum antibiotics. Intestinal amebiasis should be considered in patients with compatible symptoms and relevant exposure.

## Case report

A 30-year-old Swiss male was admitted to the emergency department at the University Hospital of Basel in June 2025 with acute onset of fever, chills and severe hemoptysis.

**Figure 1:**
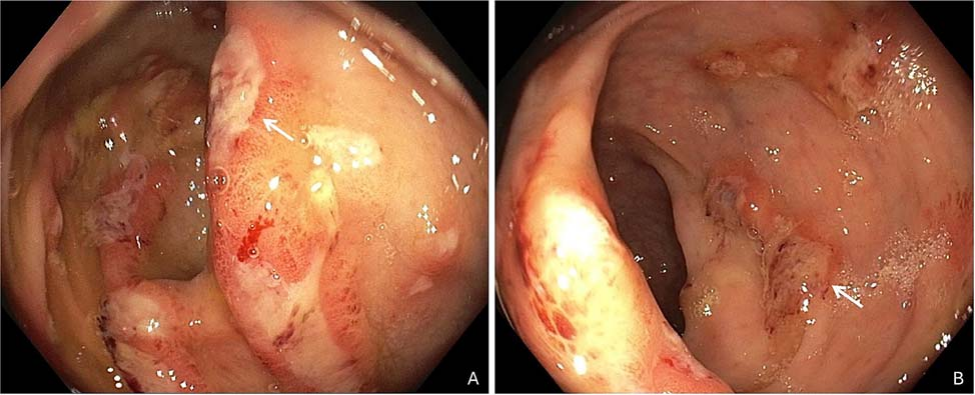
Colonoscopy with ulcerative lesions (white arrows) of the mucosa in the ascending colon (A) and right hepatic flexure (B)

The patient had no relevant medical history but had returned from rural Cambodia (district of Kampong Chhnang) two months prior to admission, where he worked at a hotel and lived in close contact to locals from November 2024 to April 2025. In January 2025, he experienced acute gastroenteritis with fever, diarrhoea and abdominal cramps which resolved quickly after treatment with ciprofloxacin (500 mg orally twice daily) for 5 days, prescribed by local physicians. Upon return to Switzerland, diarrhoea relapsed with 2–3 watery stools mixed with blood, mild abdominal cramps and 6 kg weight loss, this time without fever. In May 2025, colonoscopy revealed ulcerative lesions of the mucosa (Figure 1), with histological signs of inflammation and preserved glandular architecture. Stool culture for *Shigella* spp*.*, *Salmonella* spp*.*, *Campylobacter* spp*.* and *Yersinia* spp*.* was negative. A BIOFIRE® FILMARRAY® Gastrointestinal Panel was not performed, and histology slides could not be reviewed retrospectively. Diarrhoea improved spontaneously during the following weeks.

**Figure 2:**
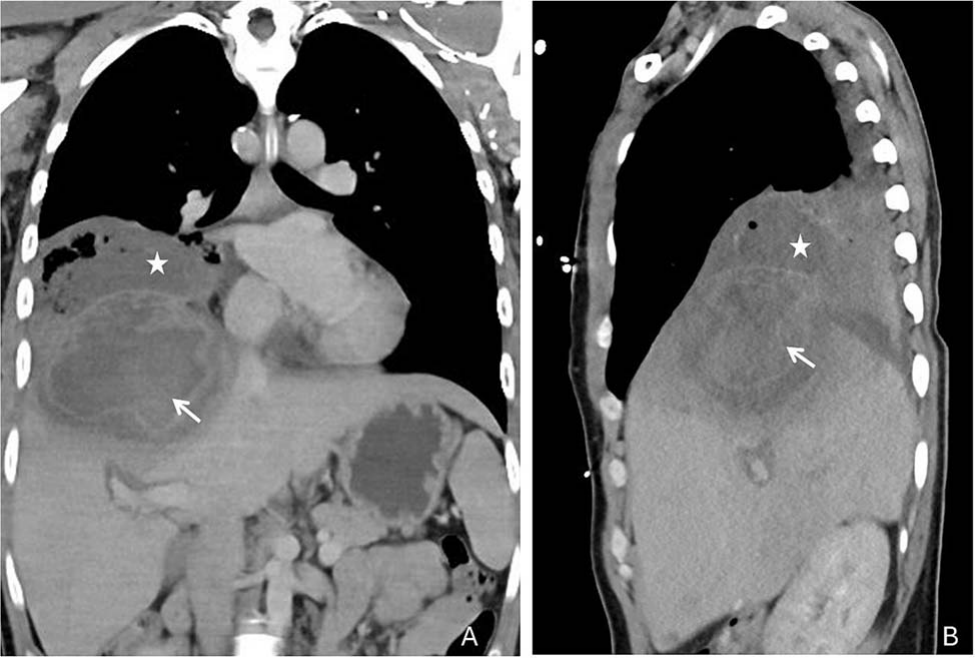
Coronal (A) and sagittal (B) reconstructions of the thoracoabdominal computed tomography revealed a peripherally contrast-enhancing collection with perifocal edema in liver segments VII/VIII (white arrows) communicating with the right broncho-pleural space (white stars)

**Figure 3:**
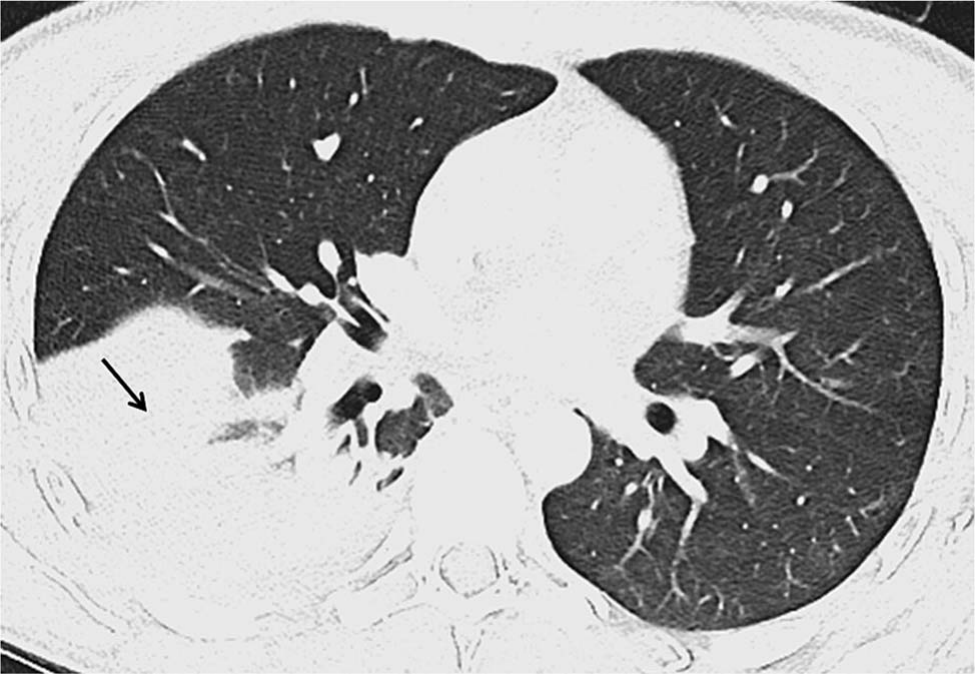
Thoracic computed tomography in axial view revealing dense infiltrates in the right lower lobe (black arrow)

Upon admission to the emergency department, the patient presented with a temperature of 40°C, respiratory distress and hemoptysis. Blood pressure was 140/90 mmHg, heart rate 120/min, respiratory rate 26/min and peripheral oxygen saturation was 91% with 4 litres of supplemental oxygen. Clinical examination revealed abdominal tenderness and decreased breath sounds over the right lung. Laboratory results showed anaemia (98 g/l, reference range 140–180 g/l) elevated leukocytes (14.3 G/l, reference range 3.5–10.0 G/l) with 85% neutrophils, CRP (140 mg/l, reference <10 mg/l), hypoalbuminemia (19 mg/l), normal liver enzymes and bilirubin. A thoracoabdominal computed tomography revealed a large abscess in liver segments VII/VIII (81 × 96 mm) with transdiaphragmatic breakthrough, pleural effusion and pulmonary infiltrates (Figures 2 and 3). Massive hemoptysis required intubation and transfer to the Intensive Care Unit. Empiric intravenous therapy with meropenem (1 g intravenous, three times daily) and metronidazole (10 mg/kg intravenous, three times daily) was initiated, targeting suspected amebiasis and pyogenic liver abscess (covering pathogens like hypervirulent *Klebsiella* spp*.* and *Burkholderia pseudomallei*).

Bronchoscopy showed signs of bronchial inflammation with discrete brownish secretion without haemorrhage. Percutaneous drainage of the liver abscess yielded anchovy-paste–like fluid. A multiplex PCR (BIOFIRE® FILMARRAY® GastrointestinalPanel), validated for stool samples applied to the drainage fluid, tested positive for *Entamoeba histolytica* with results available within 12 h. Meropenem was discontinued, and metronidazole continued, leading to rapid clinical improvement. Diagnosis of extraintestinal amebiasis with amebic liver abscess and bronchopleural involvement was confirmed by specific PCR for *E. histolytica* from both liver abscess and bronchial fluid samples, as well as by serology, with results available four days after specimen collection. Clinical follow-up in July 2025 showed complete recovery.

Infection with *E. histolytica* remains a significant global health burden, increasingly relevant beyond low- and middle-income countries due to international travel.^1^^,^^2^

Although rare, amebic liver abscess with thoracic extension is a serious complication, usually caused by diaphragmatic rupture into the pleural space. This can lead to pleuropulmonary manifestations such as empyema, lung abscess or broncho-hepatic fistula, which often require surgical drainage when conservative treatment proves insufficient. With prompt anti-amebic therapy and intervention, the prognosis is favourable.^3^^,^^4^

This case highlights the importance of considering intestinal and extraintestinal amebiasis in patients with compatible symptoms and relevant epidemiological exposure. Application of targeted molecular diagnostics enabled early pathogen identification, leading to targeted therapy within 24 h after admission. Here, BIOFIRE® multiplex PCR was successfully applied to abscess fluid, despite being validated only for stool specimen, highlighting the need for further evaluation of this off-label application.^5^
